# HOMA-IR is positively correlated with biological age and advanced aging in the US adult population

**DOI:** 10.1186/s40001-023-01448-1

**Published:** 2023-10-28

**Authors:** Haifang Yang, Rongpeng Gong, Moli Liu, Ying Deng, Xiaoyu Zheng, Tianyang Hu

**Affiliations:** 1 Medical College of Qinghai University, Xining, China; 2 Department of Cardiology, The First Branch, The First Affiliated Hospital of Chongqing Medical University, Chongqing, China; 3 School of Clinical Medicine, Chongqing Medical and Pharmaceutical College, Chongqing, China; 4 Precision Medicine Center, The Second Affiliated Hospital of Chongqing Medical University, Chongqing, China

**Keywords:** HOMA-IR, Biological age, Advanced aging, Cross-sectional study, NHANES

## Abstract

**Background:**

Insulin resistance (IR) had been reported to be associated with age; however, few studies have explored the association between IR and biological age (BA). The HOMA-IR value is a useful indicator of the extent of IR. This cross-sectional study is to explore the relationship between HOMA-IR and BA/advanced aging in the US population.

**Methods:**

This study is a cross-sectional analysis of National Health and Nutrition Examination Survey (NHANES) data. The survey comprised 12,266 people from the NHANES, and their full HOMA-IR data as well as BA data were extracted. Four multiple linear regressions were performed to analyze the association between HOMA-IR and BA, and four multiple logistic regression models were performed to analyze the association between HOMA-IR and advanced aging. In addition, trend tests and stratified analysis were performed and smoothed fitted curves were plotted to test the robustness of the results.

**Results:**

HOMA-IR was positively correlated with BA [*β*: 0.51 (0.39, 0.63)], and it was the same to advanced aging [OR: 1.05 (1.02, 1.07)], and both showed a monotonically increasing trend. The trend tests showed that the results were stable (all *P* for trend < 0.0001). The smoothed fitted curves showed that there were non-linear relationships between HOMA-IR and BA/advanced aging. And the stratified analysis indicated that the relationship between HOMA-IR and BA/advanced aging remained robust in all subgroups.

**Conclusion:**

The study suggested that HOMA-IR is positively correlated with BA and advanced aging in the US adult population, with a monotonic upward trend. This is a new finding to reveal the relationship between HOMA-IR and age from new standpoint of BA rather than chronological age (CA). And it may contribute to a better understanding of human health aging and may aid future research in this field.

## Introduction

Insulin resistance (IR) is characterized as the decrease in the effectiveness of insulin in glucose utilization or over-secreting of insulin to maintain the stability of blood glucose levels [1]. Previous studies had shown that IR contributes to the development and poor prognosis of some diseases, including diabetes [2], obesity [3], polycystic ovarian syndrome (PCOS) [4], hypertension [5], cardiovascular disease [6], kidney disease [6], Non-alcoholic fatty liver disease (NAFLD) [7], psoriasis [8], and cancers such as colon cancer [9], pancreatic cancer [10] and liver cancer [11]. Schutze et al*.* [12] conducted a cohort study including 7199 patients without diabetes, hypertension or cardiovascular disease (CVD) in 2021, they found that IR was a risk factor for hypertension (RR = 1.32, 95% CI 1.10–1.58, *P* < 0.001). In 2019, one research containing 2483 participants was exerted by Huang et al*.* [14], they asserted that obese people with high IR were at increased risk of NAFLD (OR = 3.85, 95% CI 1.87–8.36, *P* = 0.0002), so did the thin with high IR (OR = 2.52, 95% CI 1.13–5.54, *P* = 0.02). Chan et al*.* [8] conducted a 21-year follow-up cohort study encompassing 21,789 postmenopausal women, they suggested that high-level HOMA-IR participants at baseline were more likely to develop psoriasis at follow-up (HR = 1.39,95% CI 1.08–1.79, *P* = 0.011). Furthermore, an investigation enrolled 71 patients was carried out by Avilés-Jurado et al*.* [13], all patients got a head and neck squamous cell carcinoma (HNSCC) and were untreated*.* This research indicated that patients with intermediate-advanced stage HNSCC and higher HOMA-IR levels had lower disease-free survival (HR = 2.741, 95% CI 1.023–7.341, *P* = 0.045). These studies proposed a pathogenic role for HOMA-IR in physiological aspects.

Recent studies had confirmed the strong correlation between age and IR risk previously observed [14]. In a study that recruited 3796 non-diabetes participants whose age ranged from 30 to 79, Oya et al*.* [15] displayed that age was positively linked with HOMA-IR in postmenopausal women and men (*P* < 0.001). Rodríguez-Morán et al*.* [16] performed a case–control study consisting of 100 Mexican women in 2003, they showed that women with high HOMA-IR were older (*P* = 0.0004). These investigations might point to a possible connection between IR and age; however, all of them were done with a focus on chronological age (CA). CA refers to the length of time that a person experiences from birth to the present moment while BA reflects the physiological and functional performance that corresponds to a specific CA, it takes into account the factors such as time elapse, genetics, nutrition, and so on [17]. Some scholars have recently discovered that BA can better represent a person's physiological function and aging level more than CA, and BA is more consistent with an individual's "true life expectancy" [18]. In 2022, Drewelies et al*.* [19] implemented a research including 1901 elderly participants aged 70–103, they revealed that higher BA was associated with higher physician-observed morbidity (*P* < 0.05) and higher mortality (*P* < 0.05). In 2019, Kresovich et al*.* [20] carried out a prospective cohort study enrolling 2764 women, they measured participant’s baseline blood DNA methylation, and found that breast cancer had a greater chance to develop in a woman whose BA was higher than her CA. These studies exhibited that BA and CA were two different dimensions of biological concept that might differ in the risk of morbidity and mortality.

Some aging research had revealed the important role of BA. BA represented one’s physiological and functional performance [17]. In humans, aging manifested itself in various aspects such as cognitive function, physiological function, and lifespan, all of which were closely associated with BA. A study [21] of 2458 participants stated that discordant BA and CA led to cognitive and physical functional decline, especially in those who were prematurely aging (BA ≥ median, CA < median). In a study using the inflammatory aging clock to evaluate BA, Sayed et al*.* [22] considered that people with BA < CA had a lower inflammatory aging clock, meaning that they had a stronger immune system and thus lived longer. In a study including 1260 subjects aged 64 years or older, Viljanen et al*.* [23] found that the elders who had a lower BA were more likely to feel healthy and satisfied with their lives after a 20-year follow-up. People with BA < CA lived longer than those with BA > CA, with an average difference in survival probability of 1.5 years [19].

These new findings make us wonder whether there is also an internal relationship between IR and BA/advanced aging. If the HOMA-IR measured by scientific methods is higher, that is, stronger IR, will it correspond to a higher BA or advanced aging? Based on this, we design this cross-sectional study to explore the connection between HOMA-IR index and BA/advanced aging.

## Method

### Participants and study design

Assuming that HOMA-IR index is not related to BA, we use the NHANES database to screen the US population and explore the relationship between HOMA-IR index and BA through statistical analysis, hoping to fill up the gaps between IR and BA. The independent variable for this study was the HOMA-IR, it was computed by follow formula: fasting blood glucose level (FPG, mmol/L) × fasting insulin level (FINS, μU/mL)/22.5 [24]. The dependent variable was BA, which was calculated from eight biological indicators: C-reactive protein (CRP), serum creatinine, serum total cholesterol (TC), serum alkaline phosphatase (ALP), glycosylated hemoglobin (Hb1Ac), serum albumin, blood urea nitrogen (BUN), and systolic blood pressure (SBP). Covariates included marital status, ethnic, alcohol use, gender, income, smoking, Body Mass Index (BMI), Alanine transaminase (ALT); Aspartate transaminase (AST); High-density lipoprotein (HDL); and Triglycerides (TG). In this study, if BA was greater than CA, it would be defined as advanced aging. We selected data of 101,316 individuals between 1999 and 2018 from the NHANES database. Participants who were older than 20 years and finished an examination and interview from 1999 to 2018 were recruited in this study. The excluded criteria in this study were as follows: (1) Participants who were younger than 20 years. (2) Participants who lacked the variables used to calculate HOMA-IR and BA were excluded. (3) Participants who were taking medicine that affected insulin, glucose metabolism, or lipid metabolism would not be able to enter the study. (4) People who got serious diseases, such as mental disorders and cancers.

#### Fasting insulin and fasting glucose measurements

Fasting glucose: Using the Roche Cobas C311 system, an ultraviolet ray test was performed to determine the blood glucose level during the fasting state. The reagents used to detect fasting glucose were Roche product #1876899, GLU reagent kit: (1) R1 reagent (6 × 66 mL). TRIS buffer, pH 7.8, Mg^2+^ , ATP, NADP, preservative. (2) R2 reagent (6 × 16 mL). HEPES buffer, pH 7.0, Mg^2+^ , hexokinase, glucose-6-phosphate dehydrogenase, and preservative. The basic idea was that glucose would react with Adenosine-triphosphate (ATP) to form Nicotinamide adenine dinucleotide (NADH) in an environment of glucose-6-phosphate dehydrogenase and hexokinase. The concentration of NADH would be proportional to the amount of glucose present, and it might be detected using a spectrophotometer at 340 nm.

Fasting insulin: Using the Roche ELECSYS 2010 instrument and Insulin Kit (Catalog number 12017547 122 (100 determinations) Roche Diagnostics Corporation, Indianapolis, IN 46250), insulin was immunoassayed. A reactive mixture of insulin and two insulin-specific monoclonal antibodies produced chemiluminescent emission in a measuring cell. And then, photomultiplier tubes were utilized to evaluate the chemiluminescent emission. It was conceivable to use the amount of light generated as a proxy for insulin concentration. The results were decided by a master curve derived from the reagent barcode and an instrument-specific calibration curve from 2-point calibration.

#### Biological age and superannuated aging determination

The American Federation for Aging Research proposed the following criteria for biomarkers of aging. (1) They must be indicators of the rate of aging. (2) They can monitor a fundamental process that underlies the aging process but not disease effects. (3) They must be possible to test repeatedly without injuring the individual. (4) It must be effective on both humans and laboratory animals [25]. Chen et al*.* [26] referred to the BA calculation method proposed by Klemera and Doubal [18] in calculating the BA. The BA was calculated according to the formula shown below, using parameters of regression of CA on 8 biomarkers, such as slope, intercept and root mean square error. The biomarkers in this study we used were the same as in Chen’s study: SBP, BUN, CRP, Hb1Ac, serum creatinine, ALP, serum albumin, and TC (Table 1). The selected biomarkers met all the aforementioned criteria. Chen's study did not report the predictive ability of the eight biomarkers in their research. However, Levine's [27] study, which utilized 10 biomarkers and the Klemera and Doubal method to calculate biological age, demonstrated good predictive capability with an ROC value of 0.853. Among these 10 biomarkers in Levine’s study, all 8 of the biomarkers included in our research were incorporated. The biomarkers were measured as follows: (I) SBP: Averaged readings from three separate sphygmomanometer readings of the diastolic pressure at rest were used to get the systolic reading. (II) BUN: The enzymatic rate approach was utilized in conjunction with a Beckman Coulter UniCel^®^ DxC660i Synchron Clinical System and Synchron Systems Urea Nitrogen Reagent [BUNm] (Part #472482) to ascertain the level of urea nitrogen in the blood. (III) CRP: Latex enhanced nephelometry was used to estimate CRP levels, using the following reagents: (1) N Latex CardioPhase hsCRP Reagent, cat. #OQIY 21. (2) Rheumatology std SL, cat # OQKZ 13. (3) N Supplementary Reagent/Precipitation, cat #OUMU15. (4) N Diluent, cat # OUMT 65. (5) N Rx buffer, cat # OUMS65. (IV) A minimum of 1 mL of whole blood was drawn by trained staff, chilled at 2–8 °C, and sent to the lab. After a series of chemical reactions between the blood and reagents, a hemoglobin complex termed SA1c was created, and its concentration mirrored that of Hb1Ac. The reagents used for detecting Hb1Ac were as follows: Elution Buffer HSi Variant No. 1, Part # 021446 (1 × 1500 mL); Elution Buffer HSi Variant No. 2, (S) Part # 019553 (1 × 800 mL); Elution Buffer Hsi Variant No. 3, (S) Part # 019554 (1 × 800 mL); and Hemoolysis & Wash Solution, Part # 018431. (V) Serum creatinine: For the enzymatic reactions, the creatinine reagent kit [Synchron Creatinine Reagent Kit (Part #472525)] was required. As a control reagent, BIO-Liquid Rad's unassayed multiqual levels 1 and 3 were utilized. At 560 nm, a colored chromogen reflecting creatinine concentration was formed and identified. (VI) ALP was determined by Beckman Coulter UniCel^®^ DxC 800 & DxC 660i Synchron Clinical Systems and Beckman Synchron System ALP Reagent (Part #442670, 200 test/cartridge or Part #476821, 400 test/cartridge), using a kinetic rate method with 2-amino-2-methyl-1-propanol (AMP) buffer. (VII) Serum albumin: Using a timed endpoint approach, the samples were examined with Beckman Synchron System Albumin Reagent (Part #467,858) within 2–3 days of receipt. The albumin first interacted with bromcresol purple to generate a colorful product. The subsequent detection of the change in absorbance was carried out mechanically. (VIII) TC: The samples were tested by employees of the Fairview-University Medical Center, using Beckman Synchron System Cholesterol Reagent (Part #467825). At least 100 μL of samples were needed for the test. Ethylene diaminetetra acetic acid tetrasodium salt (EDTA) was the anticoagulant in use. Formic acid, sodium hydroxide, citric acid, and other substances were used in the cell wash solutions. In this study, advanced aging was defined as BA greater than CA, conversely, non-advanced aging was defined as BA less than or equal to CA.

**Table 1:** The biomarkers used to calculate biological age

	Name of biomarkers	Abbreviations
1	Systolic blood pressure	SBP
2	Blood urea nitrogen	BUN
3	Serum c-reactive protein	CRP
4	Glycosylated hemoglobin	Hb1Ac
5	Serum creatinine	/
6	Serum alkaline phosphatase	ALP
7	Serum albumin	/
8	Serum total cholesterol	TC

1 $$\mathrm{BA}=\frac{\sum_{j=1}^{m}\left({x}_{j}-{q}_{j}\right)\left(\frac{{k}_{j}}{{s}_{j}^{2}}\right)+\frac{\mathrm{CA}}{{s}_{BA}^{2}}}{\sum_{j=1}^{m}{\left(\frac{{k}_{j}}{{s}_{j}}\right)}^{2}+\frac{1}{{s}_{\mathrm{BA}}^{2}}}$$

2 $$ \begin{aligned} s_{BA}^2 = \; \frac{{\sum_{j\; = \;1}^n {\left( {\left( {{\text{BA}}_{Ei} - {\text{CA}}_i } \right) - \frac{{\sum_{i\; = \;1}^n {\left( {{\text{BA}}_{Ei} - {\text{CA}}_i } \right)} }}{n}} \right)^2 } }}{n} \\ - \; \left( {\frac{{1 - r_{\text{char }}^2 }}{{r_{\text{char }}^2 }}} \right) \times \left( {\frac{{\left( {{\text{CA}}_{{\text{max}}} - CA_{\text{min }} } \right)^2 }}{12m}} \right) \\ \end{aligned} $$

3 $${\mathrm{BA}}_{E}=\frac{\sum_{j=1}^{m}\left({x}_{j}-{q}_{j}\right)\left(\frac{{k}_{j}}{{s}_{j}^{2}}\right)}{\sum_{j=1}^{m}{\left(\frac{{k}_{j}}{{s}_{j}}\right)}^{2}}$$

4 $${r}_{char}=\frac{\sum_{j=1}^{m}\frac{{r}_{j}^{2}}{\sqrt{1-{r}_{j}^{2}}}}{\sum_{j=1}^{m}\frac{{r}_{j}}{\sqrt{1-{r}_{j}^{2}}}}$$

#### Covariate selection and definitions

The covariates selected for this study included sex, ethnic, marital status, poverty–income ratio (PIR), smoking, alcohol use, BMI, ALT, AST, HDL, and TG. The selected covariates were related to HOMA-IR and BA based on the previous studies. When a variate was introduced into the base regression model resulting in the model effect value changed by more than 10%, it would be opted as a covariate and then be adjusted in the regression models.

BMI was calculated as weight (kg)/height (m^2^) [28]. AST, ALT, HDL and TG were obtained from the laboratory data of NHANES, which were collected, processed and analyzed by trained personnel. Using the enzymatic rate method, the machine Beckman Coulter UNICEL DxC 800 & DXC 660i Synchron Clinical Systems and Beckman Synchron System AST Reagent (Part #442665, 200 tests/cartridge or #476831, 400 tests/cartridge) were applied to detect the nicotinamide adenine dinucleotide (NAD) reflecting the AST activity. The kinetic rate method using Beckman Synchron System ALT Reagent (Part #442620, 200 tests/cartridge or Part #476826, 400 tests/cartridge) was used to evaluate ALT activity in serum or plasma with a Beckman Coulter UniCel^®^ DxC 800 & DxC 660i Synchron Clinical System. HDL was detected by the Cobas 6000 Chemistry Analyzer with Roche product #04713214, HDL-C plus third-generation reagent kit. And TG was measured by the Beckman Coulter UniCel^®^ DxC 800 & DxC 660i Synchron Clinical Systems using the timed endpoint method with Beckman Synchron System Triglycerides Reagent (Part #445850). PIR provided a measure of a household's financial well-being by comparing its income to the poverty line. Ethnic: the classification of Ethnic in this study was consistent with the NHANES database, including Non-Hispanic White, Mexican American, Non-Hispanic Black, other Hispanic and other ethnics. Marital status: NHANES contained information for those who are widowed, divorced, married, never married, living with a partner, and separated, participants were grouped accordingly in this study. Smoking: In NHANES, respondents were asked whether they had smoked more than 100 cigarettes in their life. The one who admitted it but had quitted was classified as a former smoker. The individual who confessed smoking and continued to do so was classed as a current smoker. And the person who gave a denial answer was considered as never smoker [29]. Alcohol use: Based on the previous research, men who drank more than 14 drinks a week were defined as heavy drinkers, as were women who drank more than seven drinks a week. Moderate drinkers had between 3 and 14 drinks per week for men, with women having between 3 and 7. The average weekly intake for a mild drinker was fewer than three alcoholic beverages. Former drinkers often drank more than 12 drinks in the 12 months before the survey. Never-drinkers drank no more than 12 drinks in their lifetime [30].

### Statistical analysis

All data were analyzed by R.4.1.2. Weighted mean ± SD and weighted median (IQR) were used to express continuous variables with normal distributions and skewed distributions, respectively. Between-group comparisons with normal distributions were performed with *t*-test, whereas Mann–Whitney *U* tests were used for comparisons with skewed distributions. Weighted *n* (%) was applied to describe categorical variables, using chi-square test for comparison between groups. Four multiple linear regression models were constructed to explore the association between HOMA-IR and BA, while four multiple logistic regressions were built to investigate the relationship between HOMA-IR and advanced aging. The continuous variable HOMA-IR was transformed into categorical variable according to quartiles, and *P* for trend values were calculated to observe the possibility of non-linearity. Curve fitting was also constructed. This study used restricted cubic splines based on the linear regression to plot fitted smoothing curves to visually describe the relationship between the HOMA-IR and BA, and restricted cubic splines based on the logistic regression models was for the HOMA-IR and advanced aging. To obtain the best fitting of the restricted cubic spline models and prevent over-fitting, the number of knots was chosen between 3 and 8, and the selection was made for the number of knots with the lowest AIC value. Finally, the knots’ number for the restricted cubic spline based on linear regression model was 7, and 3 for restricted cubic spline based on the logistic regression model.* P* < 0.05 was regarded as statistically significant.

## Results

### Description of the basic characteristics of the population

**Fig. 1:**
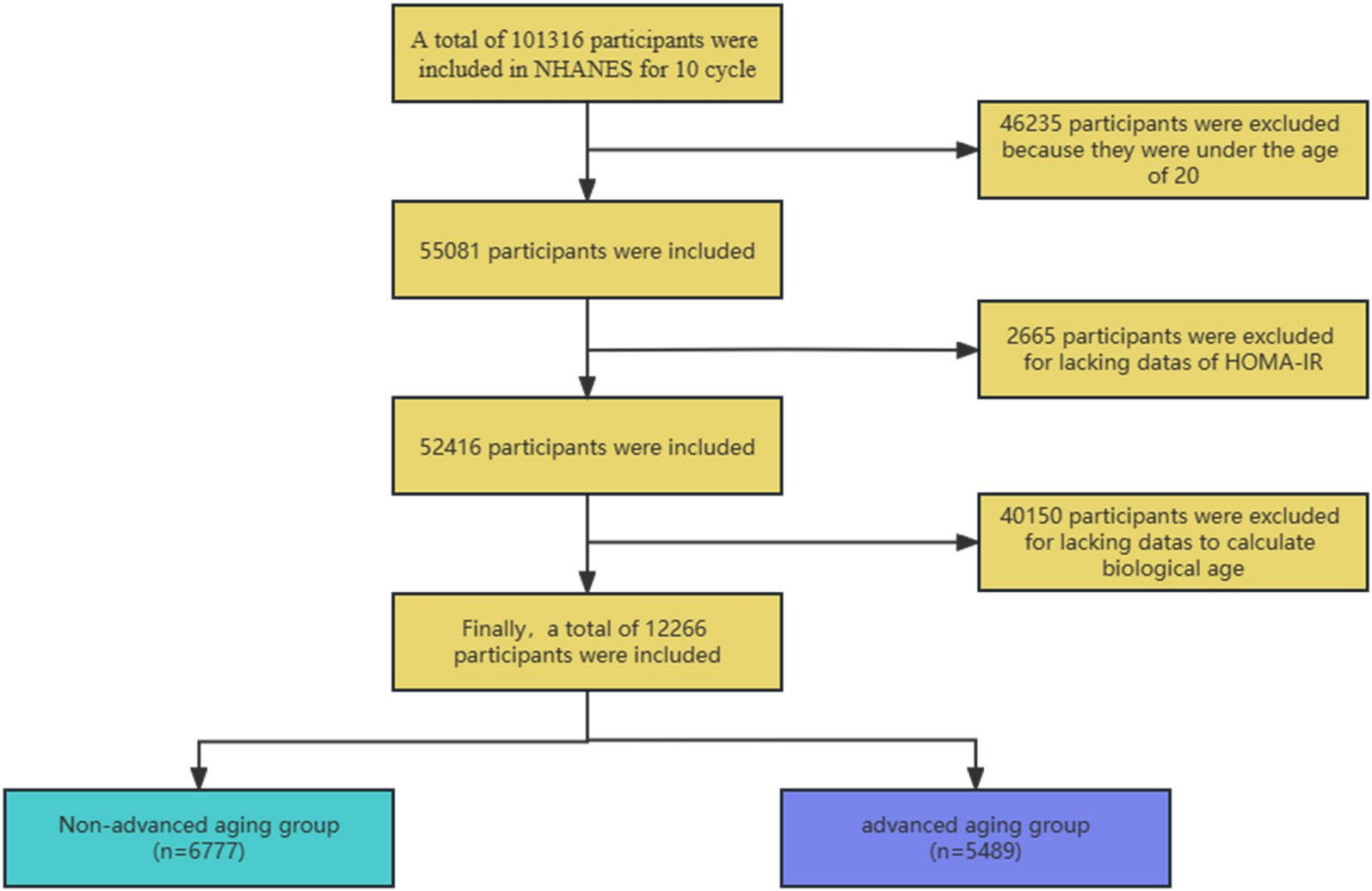
Flowchart of participant selection

From 1999 to 2018, 101,316 participants were included in NHANES. Following application of exclusion criteria, 12,266 individuals were involved in the analysis (Fig. 1). The individuals’ characteristics in both research groups are outlined in Table 2. Participants were divided into two groups based on whether or not they exhibited advanced aging, those whose BA exceeded their CA were allocated to the advanced aging group [5489 (44.7%)]. The participants’ average age is 46.4. People with advanced aging were more likely to be male, had a higher BMI (29.7 vs 27.2 kg/m^2^, *P* < 0.0001), and had lower PIR (2.9 vs 3.1, *P* < 0.0001). Among advanced aging population, mild drinkers and married people accounted for the highest proportions, 33.2% and 54.2% respectively, and were more likely to be non-Hispanic whites (67.6%). Compared with the non-advanced aging group, the advanced aging group had higher levels of AST (23.0 vs 22.0 U/L), TG (1.3 vs 1.10 mmol/L), ALT (24.0 vs 20.0 U/L) and HOMA-IR (3.9 vs 2.6), all the differences were statistically significant (All *P* < 0.001). In contrast, participants in the non-advanced aging group had higher HDL (1.4 vs 1.2 mmol/l, *P* < 0.0001). No significant difference existed between the two groups in terms of smoking.

**Table 2:** Basic characteristics of the study participants

Variable	Total (*n* = 12,266)	Non-advanced aging (*n* = 6777)	Advanced aging (*n* = 5489)	*P-*value
Age, mean ± SD	46.4 (0.3)	47.7 (0.3)	44.6 (0.4)	< 0.0001
Sex, n (%)				< 0.0001
Female	6343 (51.4)	4124 (62.2)	2219 (37.5)	
Male	5923 (48.6)	2653 (37.8)	3270 (62.5)	
PIR, mean ± SD	3.0 ± 0.0	3.1 ± 0.0	2.9 ± 0.0	< 0.0001
BMI (kg/m^2^),mean ± SD	28.3 ± 0.1	27.2 ± 0.1	29.7 ± 0.1	< 0.0001
Ethnic, n (%)				< 0.0001
Mexican American	2589 (7.8)	1397 (7.2)	1192 (8.7)	
Non-Hispanic Black	2173 (10.5)	996 (8.5)	1177 (13.0)	
Non-Hispanic White	6182 (71.5)	3647 (74.6)	2535 (67.6)	
Other Hispanic	838 (4.8)	481 (4.5)	357 (5.2)	
Other Races	484 (5.4)	256 (5.2)	228 (5.5)	
Marital status, *n* (%)				< 0.0001
Divorced	1139 (9.2)	683 (10.5)	456 (7.8)	
Living with partner	867 (7.2)	430 (6.5)	437 (8.5)	
Married	6769 (57.5)	3908 (62.0)	2861 (54.2)	
Never married	1839 (15.9)	831 (12.5)	1008 (21.0)	
Separated	385 (2.5)	229 (2.6)	156 (2.4)	
Widowed	1073 (5.8)	575 (5.9)	498 (6.1)	
Alcohol use, *n* (%)				< 0.0001
Former	2389 (16.4)	1272 (16.3)	1117 (18.2)	
Heavy	2307 (19.8)	1197 (18.9)	1110 (22.8)	
Mild	3787 (33.8)	2150 (36.8)	1637 (33.2)	
Moderate	1586 (15.0)	934 (17.0)	652 (14.0)	
Never	1631 (10.9)	902 (11.1)	729 (11.8)	
Smoking, *n* (%)				0.53
Former	3241 (25.6)	1822 (26.1)	1419 (25.0)	
Never	6445 (51.4)	3526 (51.1)	2919 (52.0)	
Now	2569 (22.9)	1423 (22.9)	1146 (23.1)	
ALT (U/L), median (IQR)	21.0 (16.0,29.0)	20.0 (16.0,26.0)	24.0 (18.0,33.0)	< 0.0001
AST (U/L), median (IQR)	23.0 (19.0,27.0)	22.0 (19.0,26.0)	23.0 (20.0,28.0)	< 0.0001
HDL (mmol/L), median (IQR)	1.3 (1.1,1.6)	1.4 (1.1,1.7)	1.2 (1.0,1.5)	< 0.0001
TG (mmol/L), median (IQR)	1.2 (0.8,1.8)	1.1 (0.8,1.6)	1.3 (0.9,2.0)	< 0.0001
HOMA-IR, mean ± SD	3.1 ± 0.0	2.6 ± 0.0	3.9 ± 0.1	< 0.0001

PIR: Poverty–income ratio; BMI: body mass index; ALT: alanine transaminase; AST: aspartate transaminase; HDL: high-density lipoprotein; TG: triglycerides; HOMA-IR: homeostasis model assessment of insulin resistance

### Univariate analyses

In this study, we analyzed the association of age, ethnic, marital status, PIR, alcohol use, smoking, BMI and some biochemical indexes with BA and advanced aging in American population by univariate linear regression and univariate logistics regression, respectively.

As shown in Tables 3 and 4. BMI, ALT, AST and TG were positively correlated with BA and advanced aging (all *P* < 0.05). PIR and HDL were positively correlated with BA, but negatively correlated with advanced aging (all* P* < 0.05). Compared with women, the BA of men was 1.9 years older, the effect size of *β* and 95%CI was 1.9 (1.3,2.5), and men had a 170% increased risk of developing advanced aging [OR: 2.7 (2.5,3.0)], all *P* < 0.0001. In terms of ethnicity, compared with Mexican-Americans, the biological ages of non-Hispanic whites, non-Hispanic blacks, other Hispanics, and other races all increased, and the non-Hispanic whites increased the most [*β* 7.7 (6.5,9.0), *P* < 0.0001], and non-Hispanic blacks were more likely to be advanced aging, with the effect value OR and 95% confidence interval of 1.3 (1.1,1.4), *P* < 0.0001. In terms of marital status, those living with a partner, married, never married, and separated all had lower biological ages compared to those who were divorced; conversely, widows had an increased BA [*β* 23.3 (21.4,25.1)], and those who never married had the highest risk of advanced aging [OR 2.3 (1.9,2.7)]. Drinking was negatively associated with BA (*P* < 0.0001), and moderate drinkers had the lowest risk of advanced aging compared with former drinkers [OR: 0.7 (0.6,0.9)]. In addition, smoking was negatively correlated with BA (*P* < 0.0001), and there was no significant difference in advanced aging among former smokers, never smokers and current smokers.

**Table 3:** Association of covariates and Biological Age

Variable	*β* (95% CI)	*P-value*
Sex		
Female	0 (ref)	
Male	1.9 (1.3,2.5)	< 0.0001
PIR	0.3 (0.0,0.6)	0.03
BMI	0.4 (0.4,0.5)	< 0.0001
Ethnic		
Mexican American	0 (ref)	
Non-Hispanic Black	5.5 (4.0,7.0)	< 0.0001
Non-Hispanic White	7.7 (6.5,9.0)	< 0.0001
Other Hispanic	3.1 (0.6,5.6)	0.02
Other Races	2.7 (0.5,4.8)	0.02
Marital status		
Divorced	0 (ref)	
Living with partner	− 13.1 (− 14.7, − 11.4)	< 0.0001
Married	− 1.2 ( − 2.6, 0.2)	0.08
Never married	− 16.2 (− 17.5, − 14.9)	< 0.0001
Separated	− 7.0 ( − 9.0, − 5.0)	< 0.0001
Widowed	23.3 (21.4, 25.1)	< 0.0001
Alcohol. Use		
Former	0 (ref)	
Heavy	− 16.3 (− 17.4, − 15.2)	< 0.0001
Mild	− 5.7 ( − 6.8, − 4.7)	< 0.0001
Moderate	− 12.6 (− 13.9, − 11.4)	< 0.0001
Never	− 5.0 ( − 7.0, − 2.8)	< 0.0001
Smoking		
Former	0 (ref)	
Never	− 8.4 ( − 9.3, − 7.4)	< 0.0001
Now	− 12.1 (− 13.0, − 11.1)	< 0.0001
ALT	0.0 (0.0,0.0)	0.05
AST	0.0 (0.0,0.1)	< 0.001
HDL	1.2 (0.4,2.0)	0.01
TG	1.9 (1.4,2.5)	< 0.0001

PIR: Poverty–income ratio; BMI: body mass index; ALT: alanine transaminase; AST: aspartate transaminase; HDL: high-density lipoprotein; TG: triglycerides

**Table 4:** Association of covariates and advanced aging

Variable	OR (95% CI)	*P-value*
Sex		
Female	1 (ref)	
Male	2.7 (2.5,3.0)	< 0.0001
PIR	0.9 (0.9,0.9)	< 0.0001
BMI	1.1 (1.1,1.1)	< 0.0001
Ethnic		
Mexican American	1 (ref)	
Non-Hispanic Black	1.3 (1.1,1.4)	< 0.001
Non-Hispanic White	0.8 (0.7,0.8)	< 0.0001
Other Hispanic	1.0 (0.7,1.2)	0.74
Other Races	0.9 (0.7,1.1)	0.32
Marital status		
Divorced	1 (ref)	
Living with partner	1.8 (1.4,2.2)	< 0.0001
Married	1.2 (1.0,1.4)	0.03
Never married	2.3 (1.9,2.7)	< 0.0001
Separated	1.2 (0.9,1.6)	0.15
Widowed	1.4 (1.1,1.8)	0.01
Alcohol use		
Former	1 (ref)	
Heavy	1.1 (0.9,1.3)	0.33
Mild	0.8 (0.7,0.9)	0.003
Moderate	0.7 (0.6,0.9)	< 0.0001
Never	1.0 (0.8,1.1)	0.60
Smoking		
Former	1 (ref)	
Never	1.1 (1.0,1.2)	0.24
Now	1.1 (0.9,1.2)	0.46
ALT	1.0 (1.0,1.0)	< 0.0001
AST	1.0 (1.0,1.0)	< 0.0001
HDL	0.5 (0.4,0.5)	< 0.0001
TG	1.3 (1.3,1.4)	< 0.0001

PIR: Poverty–income ratio; BMI: body mass index; ALT: alanine transaminase; AST: aspartate transaminase; HDL: high-density lipoprotein; TG: triglycerides

### Multivariate analysis

In this study, four multivariate linear regression models were constructed to analyze the connection between HOMA-IR and BA, and another four multivariate logistic regression models were created to investigate the relation between HOMA-IR and advanced aging, as shown in Tables 5 and 6. The beta value of the model effect value indicated that for each unit increased in HOMA-IR, there was a corresponding increase in BA. And the OR value of the effect value indicated that each unit of HOMA-IR increased, the risk of advanced aging increased correspondingly. Model 1 was not adjusted, Model 2 was adjusted for sex and ethnic, Model 3 was adjusted for sex, alcohol use, ethnic, smoking, PIR, and marital status, and Model 4 was fully adjusted for sex, PIR, marital status, alcohol use, ethnic, smoking, BMI, ALT, AST, HDL, TG. In Model 1, the effect value *β* and 95% CI were 0.8 (0.7, 1.0), which implied a 0.8-year increase in corresponding BA for each unit increase in HOMA-IR. And an effect value OR and 95% CI of 1.2 (1.1, 1.2), demonstrating that the risk of advanced aging increased by 20% for every unit higher in HOMA-IR. In Model 2, the effect value *β* and 95% CI were 0.9 (0.8, 1.0), and the effect value OR and 95% CI were 1.1 (1.1, 1.2). In Model 3, the effect value *β* and 95% CI were 0.6 (0.5, 0.7), and the effect value OR and 95% CI were 1.1 (1.1, 1.2). In Model 4, the effect value *β* and 95% CI were 0.5 (0.4, 0.6), and the effect value OR and 95% CI were 1.0 (1.0,1.1). Based on the above results, HOMA-IR was related independently with BA and advanced aging. In addition, this study also performed a trend test to further confirm the reliability of the findings. HOMA-IR was converted from continuous to categorical variables using the interquartile range. HOMA-IR-Q1 was used as the reference, with the HOMA-IRQ level increased, the increased of BA as well as the increased risk of advanced aging showed a monotonically increasing trend in all 4 models (all *P for trend* < 0.0001), which indicated that HOMA-IR was strongly connected with BA and advanced aging, and the results were stable.

**Table 5:** Association between HOMA-IR and Biological Age, Adjusted for Covariates

Variable	Model 1		Model 2		Model 3		Model 4	
	*β* (95%)	*P-value*	*β* (95%)	*P-value*	*β* (95%)	*P-value*	*β* (95%)	*P-value*
HOMA-IR	0.8 (0.7,1.0)	< 0.001	0.9 (0.8,1.0)	< 0.001	0.6 (0.5,0.7)	< 0.001	0.5 (0.4,0.6)	< 0.001
HOMA-IR group								
Q1	0 (Ref)		0 (Ref)		0 (Ref)		0 (Ref)	
Q2	2.8 (1.9,3.8)	< 0.001	3.0 (2.1,3.9)	< 0.001	1.8 (1.0,2.6)	< 0.001	2.1 (1.3,3.0)	< 0.001
Q3	5.7 (4.7,6.6)	< 0.001	6.1 (5.2,7.1)	< 0.001	4.0 (3.1,4.9)	< 0.001	4.1 (3.1,5.2)	< 0.001
Q4	9.6 (8.6,10.5)	< 0.001	10.0 (9.1,11.0)	< 0.001	7.0 (6.3,7.8)	< 0.001	6.9 (6.0,7.9)	< 0.001
* P for trend*		< 0.001		< 0.001		< 0.001		< 0.001

Model 1: Non-adjusted

Model 2: Adjusted for sex, ethnic

Model 3: Adjusted for Model2 + Marital status, PIR, smoking, alcohol use

Model 4: Adjusted for Model3 + BMI, ALT, AST, TG, HDL

**Table 6:** Association between HOMA-IR and Advanced Aging, Adjusted for Covariates

Variable	Model 1		Model 2		Model 3		Model 4	
	OR (95%)	*P-value*	OR (95%)	*P-value*	OR (95%)	*P-value*	OR (95%)	*P-value*
HOMA-IR	1.2 (1.1,1.2)	< 0.001	1.1 (1.1,1.2)	< 0.001	1.1 (1.1,1.2)	< 0.001	1.0 (1.0,1.1)	< 0.001
HOMA-IR group								
Q1	1 (Ref)		1 (Ref)		1 (Ref)		1 (Ref)	
Q2	1.5 (1.3,1.7)	< 0.001	1.4 (1.23,1.6)	< 0.001	1.4 (1.2,1.7)	< 0.001	1.2 (1.0,1.5)	< 0.001
Q3	2.0 (1.8,2.3)	< 0.001	1.9 (1.6,2.1)	< 0.001	1.9 (1.6,2.2)	< 0.001	1.3 (1.1,1.5)	< 0.001
Q4	3.2 (2.8,3.7)	< 0.001	3.0 (2.6,3.4)	< 0.001	2.9 (2.5,3.4)	< 0.001	1.6 (1.3,1.9)	< 0.001
* p for trend*		< 0.001		< 0.001		< 0.001		< 0.001

Model 1: Non-adjusted

Model 2: Adjusted for sex, ethnic

Model 3: Adjusted for Model2 + Marital status, PIR, smoking, alcohol use

Model 4: Adjusted for Model3 + BMI, ALT, AST, TG, HDL

### Curve fitting

**Fig. 2:**
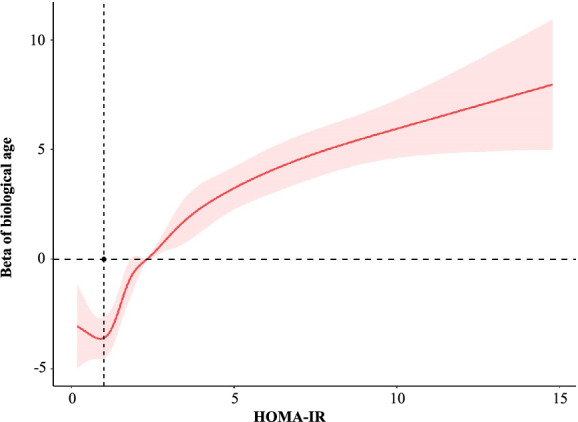
Association between HOMA-IR and biological age

**Fig. 3:**
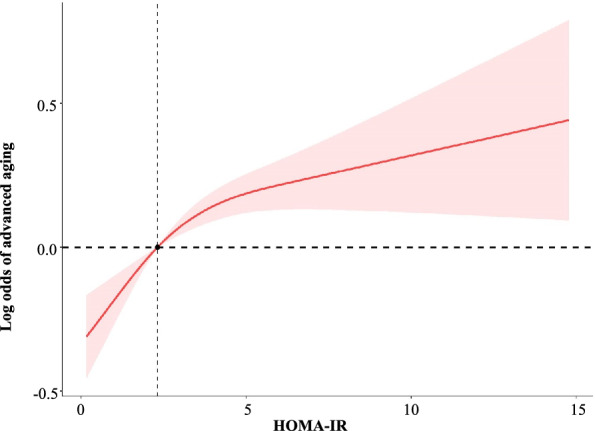
Association between HOMA-IR and advanced aging

In this study, smoothed fitted curves were drawn using restricted cubic splines based on the linear regression and logistic regression models to visually describe the shape of the relationship between HOMA-IR and BA and advanced aging, as shown in Figs. 2 and 3. The smoothed curves showed that after adjusting for adjustment for sex, ethnic, PIR, marital status, smoking, alcohol use, BMI, ALT, AST, TG and HDL, HOMA-IR level was non-linear with BA *(P for non-linear* < 0.001), and so was the risk of advanced aging (*P for non-linear* = 0.006).

### Stratified analysis

Table 7 shows the stratified associations between the HOMA-IR and BA, indicating that BA increased as the HOMA-IR increased in all subgroups. There was no significant interaction in smoke (*P* for interaction = 0.4) and ethnic (*P* for interaction = 0.1), while significant interactions were detected in sex (*P* for interaction < 0.0001), BMI (*P* for interaction < 0.001) and alcohol use (*P* for interaction = 0.03). In addition, Table 8 demonstrates that the rise in HOMA-IR was accompanied by a rise in advanced aging in all subgroups. There was no significant interaction in smoke (*P* for interaction = 0.3), ethnic (*P* for interaction = 0.5) and alcohol use (*P* for interaction = 0.2), while significant interactions were observed in sex (*P* for interaction = 0.001) and BMI (*P* for interaction = 0.02). And a stronger association between the HOMA-IR and advanced aging was detected in people who had a BMI under 18.5.

**Table 7:** Stratified associations between HOMA-IR and Biological Age, Adjusted for Covariates

Characteristic	No. of participants	*β* (95% CI)	*P*	*P* for interaction
Sex				< 0.0001
Female	6343	0.8 (0.6, 1.1)	< 0.0001	
Male	5923	0.5 (0.3, 0.6)	< 0.0001	
Smoke				0.4
Former	3241	0.5 (0.2, 0.8)	< 0.001	
Never	6445	0.5 (0.3, 0.7)	< 0.0001	
Now	2569	0.8 (0.5, 1.0)	< 0.0001	
BMI				< 0.001
< 18.5	181	0.7 ( − 2.6, 4.0)	0.6	
≥ 18.5, < 24	2626	1.2 (0.4, 2.0)	0.004	
≥ 24, < 28	3509	1.4 (1.0, 1.8)	< 0.0001	
≥ 28	5780	0.5 (0.4, 0.6)	< 0.0001	
Ethnic				0.1
Mexican American	2589	0.5 (0.3, 0.8)	< 0.001	
Non-Hispanic Black	2173	0.5 (0.3, 0.8)	< 0.001	
Non-Hispanic White	6182	0.6 (0.4, 0.7)	< 0.0001	
Other Hispanic	838	0.3 (0.0, 0.5)	0.1	
Other Race	484	1 (0.5, 1.6)	< 0.001	
Alcohol use				0.03
Former	2389	0.3 (0.2, 0.5)	< 0.0001	
Heavy	2703	0.5 (0.3, 0.7)	< 0.0001	
Mild	3787	0.8 (0.6, 1.1)	< 0.0001	
Moderate	1586	0.8 (0.4, 1.2)	< 0.001	
Never	1631	0.6 (0.3, 1.0)	< 0.001	

Model adjusted for sex, BMI, smoke, ethnic, alcohol use, mar, PIR, ALT, AST, TG, HDL

In each case, the model is not adjusted for the stratification variable

**Table 8:** Stratified associations between HOMA-IR and Advanced Aging, Adjusted for Covariates

Characteristic	No. of participants	OR (95% CI)	*P*	*P* for interaction
Sex				0.001
Male	6343	1.1 (1.0,1.1)	< 0.001	
Female	5923	1.1 (1.1,1.1)	< 0.0001	
Smoke				0.3
Former	3241	1.1 (1.0,1.1)	< 0.001	
Never	6445	1.1 (1.0,1.1)	0.002	
Now	2569	1.1 (1.1,1.2)	< 0.0001	
BMI				0.02
< 18.5	181	1.3 (0.5, 3.2)	0.5	
≥ 18.5, < 24	2626	1.2 (1.1,1.4)	< 0.001	
≥ 24, < 28	3509	1.1 (1.0,1.1)	0.01	
≥ 28	5780	1.1 (1.0,1.1)	< 0.0001	
Ethnic				0.5
Mexican American	2589	1.1 (1.0,1.2)	< 0.001	
Non-Hispanic Black	2173	1.1 (1.0,1.1)	0.01	
Non-Hispanic White	6182	1.1 (1.0,1.1)	< 0.0001	
Other Hispanic	838	1.2 (1.1, 1.3)	0.004	
Other Race	484	1.0 (0.9, 1.2)	0.6	
Alcohol use				0.2
Former	2389	1.0 (1.0,1.1)	0.1	
Heavy	2703	1.1 (1.0,1.1)	0.001	
Mild	3787	1.1 (1.0,1.1)	0.01	
Moderate	1586	1.1 (1.0,1.2)	0.01	
Never	1631	1.1 (1.0,1.2)	0.01	

Model adjusted for sex, BMI, smoke, ethnic, alcohol use, mar, PIR, ALT, AST, TG, HDL

In each case, the model is not adjusted for the stratification variable

## Discussion

This study is the first to report that HOMA-IR was positively correlated with BA and advanced aging after adjusted for covariates. Previous studies mostly explored the association between IR and age based on CA. However, there is a lack of relevant studies on the association between IR and BA. In this investigation, we examined the linkage between the two, which might highlight the connection among the changes in physiological function and IR in the process of human aging and could more accurately reflect the linkage between IR and age and advanced aging. We also conducted a trend test and stratified analysis, which proved that the results were robust. And the smooth fitting curves showed that HOMA-IR was non-linear with BA and advanced aging, both showed a monotonically increasing trend.

Some studies suggested that IR might be related to oxidative stress as well as to telomere length. Oxidative stress played an important role in the etiologic mechanism of IR. The excessive presence of oxidants was intricately linked to the multifaceted origin of insulin resistance, particularly in skeletal muscle tissue [31]. Superoxide was thought to stimulate mitogen-activated protein kinases (MAPKs) through a potential intracellular signaling mechanism, according to certain studies [32–34]. The MAPK activation pathway followed the same conserved three-kinase module and consisted of MAPK, MAPK kinases (MAPKK, MKK, or MEK) and MAPK kinase kinases (MAPKKK, MEKK). As a subtype of MAPK, p38MAPK was activated by its threonine and tyrosine residues, which would be dephosphorylated under various stimulations [35], then blocked insulin signaling factors involved in GLUT-4 translocation regulation, thereby reducing the ability for insulin-dependent glucose transport activity and resulting in IR [31]. Higher concentration of the maker of oxidative stress could be detected in people with IR compared to the others who did not have IR [36]. It was also widely recognized that oxidative stress was a significant factor contributing to aging [37]. Oxidative stress induced activation of MAPK cascades, leading to activation of extracellular signal-related kinases (ERKs), c-jun NH2-terminal kinases (JNKs) and p38MAPK, resulting in apoptosis and cell death. The prevention of oxidative stress focused on the aforementioned pathway could be a significant strategy that facilitates the extension of life [38]. Meanwhile, some researchers found that HOMA-IR was associated with telomere length [39]. Short telomere would down the function of the pancreas’ beta cell in mice and played an important role in diabetes pathogenesis [40]. People who were born with a short leucocyte telomere length are more like to get IR in their life, for their impaired glucose homeostasis [41]. In addition, telomere length was correlated with levels of markers of oxidative stress during aging [42], telomeres were more susceptible to oxidative stress than whole chromosomes [43], and the repair of oxidative damage in telomeric DNA was comparatively less efficient than in other regions of the chromosome, resulting in the acceleration of telomere shortening under conditions of oxidative stress, leading to the acceleration of aging [44]. Recently, high levels of biomarkers of oxidative stress were detected in the urine of people whose BA were older than their CA [45]. These studies might provide explanations for our findings. We speculated that oxidative stress as well as telomere length which were associated with the level of HOMA-IR might co-influence BA in somehow during aging, contributing to advanced aging.

Some research found that the occurrence of IR could be attributed to the dysfunction of insulin signaling pathways, including the Phosphoinositide 3-kinase/Protein Kinase B /Mammalian Target of Rapamycin (PI3K/Akt/mTOR) pathway [46]. mTOR was an effector protein downstream of PI3K/PKB in the insulin PI3K/Akt/Glycogen Synthase Kinase 1 (GSK-1) signaling pathway and possessed serine/threonine kinase activity [47]. Long-term high-fat, high-sugar diets, obesity, and nutrient excess activated mTOR, which activated its downstream effector proteins, P70 ribosomal protein S6 kinase 1 (p70S6K1) and eukaryotic translation initiation factor 4E-binding protein 1 (4E-BP1). PI3K/Akt activity was negatively regulated by phosphorylated p70S6K1, which suppressed insulin receptor substrate tyrosine phosphorylation and caused IR [48]. mTOR also played an important role in aging pathways. The manipulation of genes within the insulin-signaling system, which was recognized for its ability to modify nutrient sensing, had been demonstrated to increase the longevity of different species. The extension of life could be achieved by inhibiting the TOR signaling system through the modification of gene expression within this nutrient-sensing pathway, which was conserved across several organisms ranging from yeast to humans [49]. It is worth mentioning that there was a significant increase in lifespan, around fivefold, observed in C. elegans double mutants that possessed mutations in genes associated with both the TOR and insulin signaling pathways [50]. Since then, additional studies in flies, worms, and rodents have demonstrated that inhibiting the insulin signaling pathway prolongs life [51]. These studies might help to explain our findings.

The BA of individuals of the same CA varied. Compared with CA, BA could reflect one’s physiological function accurately. In this study, we calculated BA based on the eight biomarkers as mentioned above. These biomarkers could represent BA better. Some researchers confirmed that high level of CRP was associated with one’s physical and cognitive function [52], and CRP was a predictor of healthy aging [53], people who felt younger than their CA had a lower CRP value [54]. People who got faster aging (BA > CA) were more likely to have higher total cholesterol and glycohemoglobin, conversely, people whose biological ages were younger than their chronological ages had higher albumin [55]. ALP was a biomarker of age that was positively related to age [56]. SBP and BUN were proved to be excellent aging biomarkers for calculating BA [57]. Compared with studies that used a single biomarker to calculate BA, this study used eight biomarkers to calculate BA; the results would be more representative. Moreover, these eight biomarkers were more economical and easily available than single biomarkers such as DNA methylation. What we found in this study that HOMA-IR was positively associated with BA/advanced aging might reveal the real relationship between IR and age/aging.

The clinical value of our study is as follows: (1) to our knowledge, this is the first to investigate the relationship between HOMA-IR and BA/advanced aging, in a big sample of US adults. (2) These findings may aid future research on IR and BA and aging. (3) Aging is an important cause for many disorders [58], such as Alzheimer's disease (AD) [59], Parkinson′s disease [60], and cancer [61]. Expanding our understanding of the biological processes that promote rapid aging may result in efficient interventions. For example, medication therapies that postpone aging may have significant clinical and societal health implications. Metformin, the most commonly prescribed insulin-sensitizing agent currently used in the clinical practice, is always prescribed to individuals with IR [62]. A study found that male mice's longevity and health span were increased by long-term metformin administration (0.1% w/w in diet), which began in middle age [63]. Mohammed et al*.* [64] concluded that despite evidence of anti-aging advantages, the claim that metformin lengthens lifespan was still debatable. However, metformin could increase health span, prolonging the amount of time spent in excellent health, by lowering early mortality linked to a number of disorders, such as diabetes, cardiovascular disease, cognitive decline, and cancer. Our findings may provide a basis for future efforts to reduce the incidence of IR through anti-aging treatments.

The data of this study were obtained from NHANES database, which has a relatively large sample size and can better reduce the problem of sample bias. However, there are also some limitations to this study. The confounders in this cross-sectional study had been controlled for, but there might be unidentified covariates. Besides this study was conducted in adults and did not involve people under the age of 20, more validation studies are needed in this segment of the population in the future. Last, this study found a positive association between HOMA-IR and BA/advanced aging, but the causal relationship could not be determined. Further cohort studies are needed to investigate the causal relationship between HOMA-IR and BA/advanced aging.

## Conclusion

In conclusion, the results of this cross-sectional study suggest that HOMA-IR levels are positively associated with BA and advanced aging. The increased HOMA-IR levels implied an increased BA and an increased risk of advanced aging. The results might supplement the research on IR and age/aging.

## Data Availability

All data are from NHANES database (https://wwwn.cdc.gov/nchs/nhanes/Default.aspx). Data in this study will be made available on request by contact with the corresponding author.

## Abbreviations

SBP: Systolic blood pressure
BUN: Blood Urea Nitrogen
CRP: Serum C-reactive protein
Hb1Ac: Glycosylated hemoglobin
ALP: Serum alkaline phosphatase
TC: Serum albumin and serum total cholesterol
PIR: Poverty–income ratio
BMI: Body mass index
ALT: Alanine transaminase
AST: Aspartate transaminase
HDL: High-density lipoprotein
TG: Triglycerides
HOMA-IR: Homeostasis model assessment of insulin resistance
